# Surgical exclusion of an idiopathic saccular aneurysm in the left main trunk of the coronary artery

**DOI:** 10.1007/s00595-021-02246-0

**Published:** 2021-02-19

**Authors:** Naoki Tadokoro, Satsuki Fukushima, Yusuke Shimahara, Tetsuya Saito, Naonori Kawamoto, Hideyuki Shimizu, Tomoyuki Fujita

**Affiliations:** 1 Department of Cardiovascular Surgery, National Cerebral and Cardiovascular Center, 6-1 Kishibeshimmachi, Suita, Osaka 564-8565 Japan; 2 Department of Cardiovascular Surgery, Keio University School of Medicine, Shinjuku-ku, Tokyo, Japan

**Keywords:** Saccular coronary artery aneurysm, Left main trunk, Surgical intervention

## Abstract

**Purpose:**

A coronary artery aneurysm (CAA) can result in critical cardiac events such as thromboembolic complications or rupture. A saccular CAA located in the left main trunk (LMT) is the most critical form of this pathology and its surgical repair is challenging. We conducted this single-center study to review the surgical outcomes of patients with a saccular CAA in the LMT.

**Methods:**

Between May, 2012 and June, 2020, five patients with a saccular CAA in the LMT underwent surgery at our center. The median age at operation was 66.5 (59.7–69) years and the median diameter of the CAA was 13.0 mm (IQR 11–14 mm).

**Results:**

The CAA was fully excluded by patch closure of the LMT orifice and direct closure of the distal LMT, supplemented by coronary artery bypass grafting with the exclusive use of arterial conduits. There was no in-hospital mortality, although one patient suffered graft spasm-related myocardial infarction with complete recovery. Post-operative angiography showed a fully excluded LMT in all patients. There was no mortality or adverse cardiac events during follow-up.

**Conclusions:**

Our surgical policy for CAA in the LMT is feasible and safe; however, coronary blood flow is dependent on reliable bypasses.

## Introduction

In 2020, the coronary artery aneurysm (CAA) registry reported the global prevalence of CAA as 0.35% [1]. The vessel affected most frequently by a CAA was the left anterior descending artery (LAD, 48.6%), followed by the right coronary artery (RCA; 31.8%) and the left circumflex artery (LCX, 28.1%). However, the left main trunk (LMT) accounted for the location of only 5.3% CAAs. CAAs are defined as local dilatation in a coronary artery to 1.5 times larger than an adjacent healthy reference segment. They are caused by atherosclerosis or other pathologies such as congenital anomalies, inflammatory disease, and iatrogenic injury [2–4]. CAAs can cause critical cardiac events such as aneurysm rupture, coronary artery thromboembolism, and myocardial ischemia [5–7]. Mural thrombus is formed, potentiating the deposition of additional thrombus within the aneurysmal segments, finally resulting in the distal embolization of thrombus and acute myocardial infarction. These clinical manifestations would be most critical in a CAA located in the LMT. Importantly, because of their rarity, the indications and optimal treatment for CAAs, especially those located in the LMT have not been fully established.

There are two interventional treatment options: catheter-based and surgical CAA exclusion. While the catheter approach is minimally invasive, it is associated with many complications [7] In contrast, surgical exclusion techniques are poorly established with various surgical procedures having been reported [8, 9]. In our surgical unit, CAAs of the LMT have been fully excluded by resection or ligation of their inflow and outflow, the interrupted perfusion being supplemented by coronary artery bypass grafting (CABG). In this study, we reviewed the surgical procedures for CAA of the LMT and their outcomes to establish their feasibility and safety.

## Methods

### Patients and data collection

A search of our institutional surgical database yielded five patients who underwent surgical exclusion of idiopathic CAA located in the LMT in the National Cerebral and Cardiovascular Center Hospital between May, 2012 and June, 2020. Patients with CAA of other causes, such as congenital anomaly, inflammatory disease, or iatrogenic injury, were excluded from the study cohort. Medical charts, operation reports, and referral letters for the five patients were reviewed to extract selected data, including major adverse cerebrovascular and cardiac events. The five patients all completed follow-up for a median of 31 months (interquartile range, IQR 29–33). Data were collected in July, 2020. All patients gave written informed consent to surgery and the use of their data for diagnostic and research purposes, prior to surgery. This study was performed with the approval of the institutional review board (M30-026).

### Diagnosis and indications for surgery

**Fig. 1:**
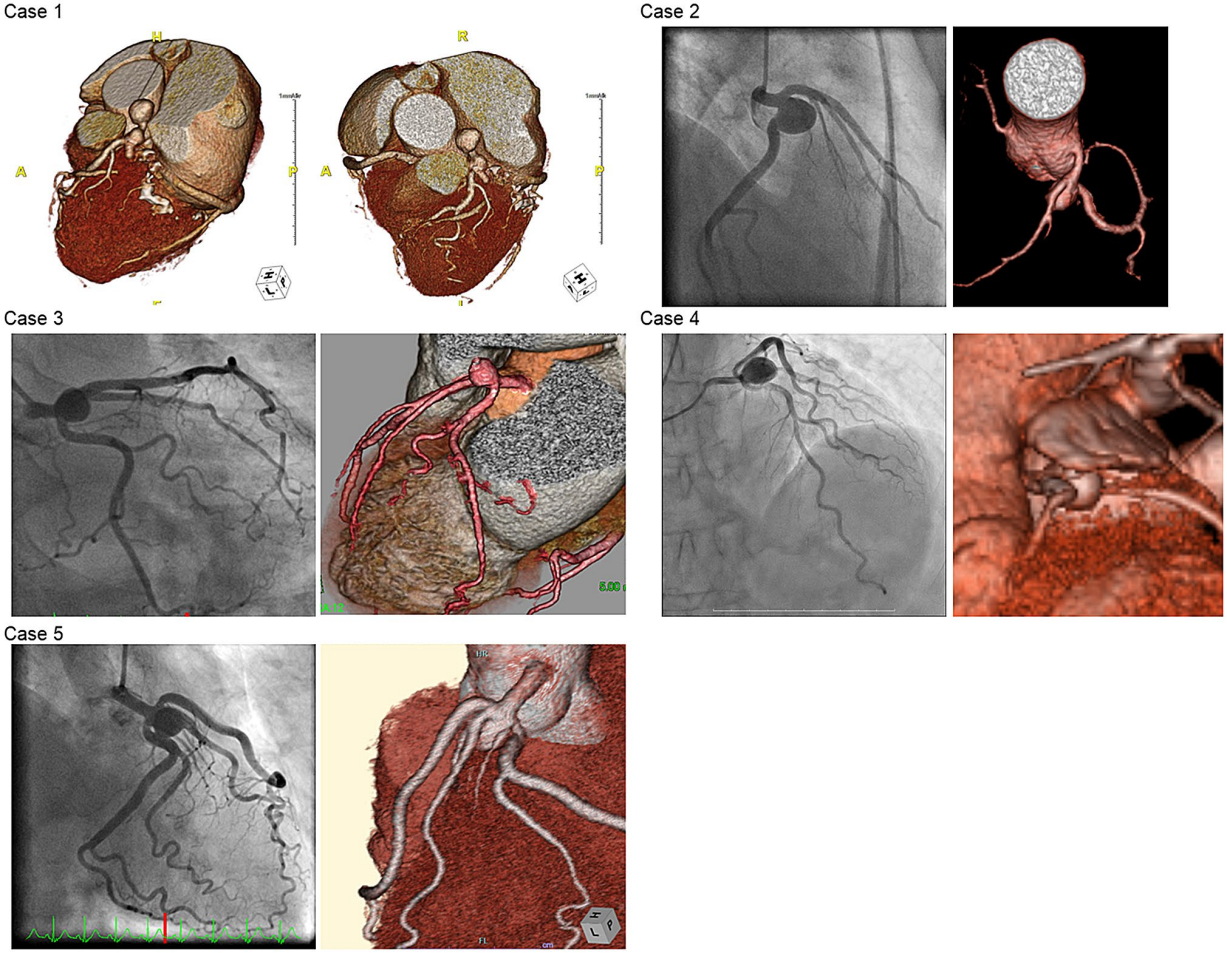
Radiological and computed tomography (CT) findings of the five patients with saccular coronary artery aneurysms

Saccular-shaped coronary artery aneurysms were diagnosed incidentally in all patients, by fluoroscopy or computed tomography (CT) coronary angiograms performed for other purposes; namely, investigation of chest pain or coronary artery disease in two patients and of arrythmia in the other three (Table 1). Surgical repair was indicated for symptomatic patients with evidence of emboli from the aneurysm to the distal coronary artery, leading to myocardial ischemia; and for patients with CAA enlargement as documented by serial angiographic measurement. In Patient 1, the main indication was a newly formed saccular aneurysm and coexistent coronary artery stenoses in the left circumflex artery (LCX); in patient 3, it was symptoms related to coronary ischemia; and in patients 2 and 4, the size of the CAA (diameter greater than 10 mm) was determined by the cardiac team to represent risk of rupture. In patient 5, the main indication of surgery was rapid expansion (1.4 mm/year) of the aneurysm (Fig. 1).

**Table 1:** Clinical characteristics of the five patients and the surgical procedures performed

Pt	Age	Sex	CAA location	CAA size (mm)	Coronary artery disease	CAA discovery	Symptoms	Pre-operative LVEF	CAA procedure	CABG procedure	Cross clamp	Post-operative LVEF
1	69	F	LMT × 2	14, 9	1-VD (LCX)	Incidental (During investigation for coronary artery disease)	None	59%	LMT ostium patch closure (from inside Ao) LAD proximal ligation	CABGx3 (RITA-LAD, LITA-Dx, LITA-RA-OM)	Yes	65%
2	53	F	LMT bifurcation*	13	No	Incidental (During investigation for arrhythmias)	Progressive dyspnea	64%	LMT ostium suture closure (from outside Ao) LAD and LCX proximal ligation	CABGx4 (LITA-LAD#7, Ao-RA-LAD#6, Ao-RA-HL, Ao-SVG-PL)	Yes	65%
3	62	F	LMT bifurcation*	8	No	Angina	Chest pain, Progressive dyspnea	62%	LMT ostium patch closure (from inside Ao) LAD, LCX orifice suture closure	CABGx3 (LITA-LAD, Ao-RA-Dx-PL)	Yes	65%
4	69	F	LMT bifurcation*	16	No	Incidental (During investigation for arrhythmias)	None	64%	LMT ostium patch closure (from inside Ao) LAD, LCX orifice suture closure	CABGx3 (LITA-LAD, Ao-RA-Dx-PL)	Yes	65%
5	66	F	LMT bifurcation*	11	No	Incidental (During investigation for arrhythmias)	None	51%	LMT ostium patch close (from inside Ao) LAD, LCX orifice suture close	CABGx4 (LITA-LAD, Ao-RA-Dx-OM-PL)	Yes	45%

*1-VD* single-vessel disease, *Ao* ascending aorta, *AV* atrioventricular branch, *CABG* coronary artery bypass grafting, *Dx* diagonal branch, *ECG* electrocardiogram, *F* female, *LAD* left anterior descending artery, *LCX* left circumflex artery, *LITA* left internal thoracic artery, *LMT* left main trunk, *M* male, *OM* obtuse marginal branch, *PL* posterolateral branch, *RA* radial artery, *RCA* right coronary artery, *SVG* saphenous vein graft, *TAR* total arch replacement

LMT bifurcation: CAA located close to or at the bifurcation of the LAD and LCX

### Surgical procedures

**Fig. 2:**
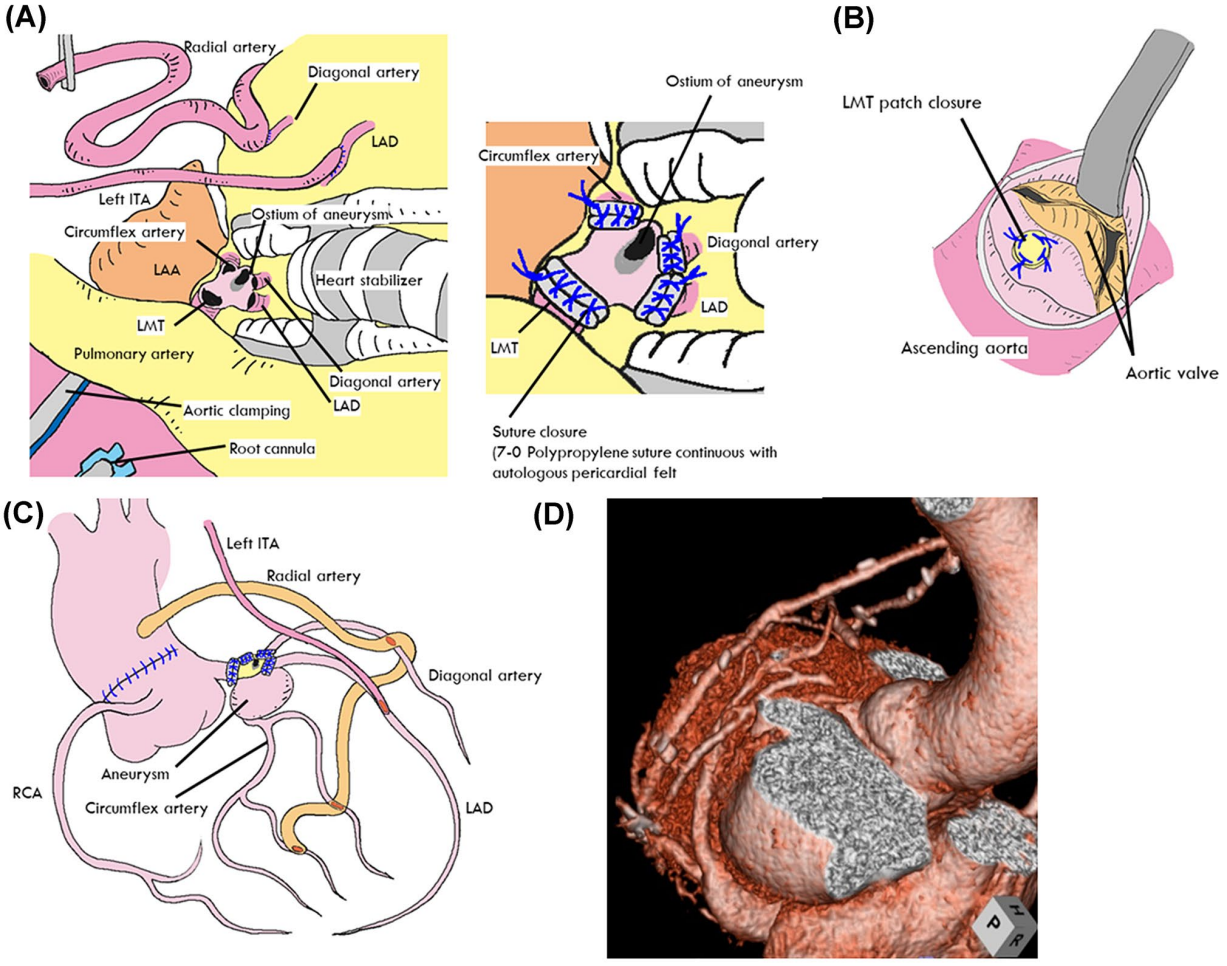
Surgical schema for the left main trunk (LMT) aneurysm (Patient 3). **a** The roof of the aneurysm was excised and the outflow of the aneurysm or orifice of the left anterior descending artery (LAD) and/or left circumflex (LCX) were sutured closed from the inside of the aneurysm. **b** The ascending aorta was incised to close the orifice of the LMT by autologous pericardial patching. **c** Flow to compensate for the interrupted coronary artery branches was established by coronary artery bypass grafting (CABG). **d** The postoperative contrast CT

The aim of surgery was to exclude the aneurysm completely, by ligation or division of its proximal inflow and distal outflow. Surgery was performed via a median sternotomy approach under induced cardiac arrest with full cardiopulmonary bypass. The roof of the aneurysm was excised and the outflow of the aneurysm or orifice of the LAD and/or LCX was closed from the inside of the aneurysm with polypropylene sutures (7–0 Prolene; Ethicon, Johnson & Johnson, Somerville, NJ, USA). Subsequently, the ascending aorta was incised to close the orifice of the LMT by autologous pericardial patching (Fig. 2). Flow to compensate for the interrupted coronary artery branches was established by coronary artery bypass grafting (CABG), performed exclusively using arterial conduits such as the internal thoracic artery (ITA) or radial artery (RA) after exclusion of the aneurysm in all patients. RA grafts were used for either individual grafts from the aorta or composite grafts, such as a Y-graft in which the RA was connected to the side of the ITA. In Patient 2, it was judged that the LITA-LAD flow was insufficient, and the RA was anastomosed to the LAD proximally from the LITA anastomosis site. All grafts were contrast-enhanced after surgery. The saphenous vein (SVGs) was used to supplement the arterial conduits. Postoperatively, all patients took aspirin 100 mg per day until the last follow-up.

### Statistical analysis

Normally continuous variables without normal distribution are presented as medians (interquartile range (IQR)) and categorical variables, as frequencies and percentages. Statistical analyses were performed using R 3.4.3 (R Development Core Team (2017), R: A language and environment for statistical computing; R Foundation for Statistical Computing, Vienna, Austria).

## Results

### Patients’ characteristics

The subjects of this single institutional case series were five patients who underwent surgery for idiopathic saccular CAA located in the LMT. None of the patients had a history of myocardial infarction related to coronary artery disease or thrombus in the CAA. Four patients (80%) had a single CAA, whereas one had multiple CAAs (Patient 1). The median diameter of the CAA was 13.0 mm (IQR 11–14 mm). Coronary angiography identified major coronary artery stenosis in one patient (20%) with single-vessel disease.

### Clinical outcomes post-surgical CAA exclusion

None of the patients had ST changes in their ECGs or new akinetic lesions on transesophageal echocardiography intraoperatively and none required mechanical circulatory support after being weaned off cardiopulmonary bypass. There was no in-hospital or 30-day mortality in the study cohort. All patients were discharged home without any postoperative complication-related ongoing disorders. One patient suffered perioperative myocardial infarction associated with spasms of the RA conduit (Patient 5). In this patient, the RA conduit was used to bypass from the ascending aorta to the diagonal, obtuse marginal, and posterolateral branches sequentially. About 2 h after transfer to the ICU, the ST segment became elevated suddenly in the V2–V4 leads with marked deterioration in the hemodynamic state. Emergency coronary angiography showed spastic occlusion of the RA conduit, into which nitroglycerin was injected, subsequent to which the ST elevation and RA spasm resolved gradually. Angiography showed a fully excluded LMT and echocardiography on day 7 showed normal LV function without wall motion abnormalities. This patient was still symptom free at their most recent follow-up, 2 years after surgery. No other patients suffered perioperative coronary artery-related events. Post-operative CT coronary angiography showed a fully excluded LMT and patent graft conduits in all these patients. There were no coronary artery-related events throughout follow-up.

### Pathological examination of the CAAs

The resected CAAs from four patients (patients 2–5) were examined pathologically. In all cases, the aneurysm was confirmed pathologically to be a true aneurysm with all arterial wall layers; namely, the intima, media, and adventitia. Four patients (patients 2–5) had nonspecific aneurysmal dilatation of the affected coronary artery.

## Discussion

We reviewed a single institution series of five patients with idiopathic CAA treated by surgical exclusion with CABG. The CAA exclusion was achieved by surgical closure of the proximal and distal ends of the LMT with CABG for the LAD and LCX territories using arterial conduits exclusively. Apart from the one patient with graft conduit spasm-related myocardial infarction, who had full functional recovery, none suffered perioperative complications.

CAAs can be treated by medical management, surgical resection, or stent placement; however, the optimal treatment of a CAA of the LMT is controversial and depends on the clinical situation. In general, surgery is appropriate for symptomatic patients with obstructive coronary artery disease, evidence of embolization leading to myocardial ischemia, or progressive enlargement of their CAA, or a CAA at risk of rupture. It could be asserted that our surgical approach, as described in this study, is too aggressive because the left main orifice is closed, and coronary perfusion of the left ventricle is, thus, permanently dependent on the bypassed grafts. Bypassed conduits may be insufficient to meet coronary perfusion demands, particularly in the early postoperative period. Moreover, disease in the bypassed conduit could limit cardiac function and cause adverse events in the long term. However, myocardial ischemia related to insufficient conduit flow did not develop in any of the patients postoperatively, although one suffered sudden spasms of the RA conduit to the LCX territory. These data indicate that bypass grafts can provide sufficient blood flow to the left ventricle after full closure of the LMT. We used arterial conduits exclusively, to achieve long-term functionality of the bypassed grafts. Further follow-up is needed to establish the safety and efficacy of this approach.

We consider that incomplete exclusion of the CAA would cause several complications in the long term, including thrombus formation in the remaining CAA endothelium or in situ recurrence of the CAA. Because most idiopathic CAAs take the form of a single aneurysm in the proximal coronary arteries, full surgical exclusion achieves full resolution of this pathology. In contrast, a CAA associated with inflammatory disease, such as Kawasaki disease (Tadokoro et al. [10]), often involves the formation of multiple aneurysms. In such patients, surgical exclusion would need more careful consideration. In fact, rupture of a CAA associated with Kawasaki disease is reported to be rare, indicating that interventional treatments are unnecessary.

Published reports describe the following surgical options for managing LMT aneurysm: CABG alone; CABG and ligation of both ends of the aneurysm [8]; CABG, ligation of the distal side of the aneurysm, and left coronary ostium closure [11, 12]; CABG and repair [13]; and CABG and polytetrafluoroethylene (PTFE)-covered stent implantation [14, 15]. There have been no reports of death associated with any of these surgical procedures, which are, therefore, considered safe and feasible. We chose the surgical procedure described here based on our experience of treating two contiguous aneurysms in the LMT. The advantages of our surgical procedure are as follows: the approach does not require cutting of the pulmonary artery trunk; (ii) under the infusion of retrograde cardioplegia, it is possible to suture all the feeding vessels closed from inside the aneurysm by excising its anterior wall; and the resultant absence of blood flow in the LMT prevents recurrence of an aneurysm. However, because procedures on all left coronary arteries require bypass grafts, establishing reliable bypass and good postoperative management are essential. Even for branched CAAs, resection/ligation with additional CABG may be suitable. For LMT CAAs, especially those located close to or at the bifurcation of LAD and LCX, ligation of both ends, in particular, of the inflow of the CAAs is technically challenging. Conversely, patch closure of the LMT orifice is not technically demanding. More importantly, ligation of both ends could leave residual aneurysmal pathology in the coronary artery.

There is still no consensus on the treatment of CAA; however, given that surgery is performed to prevent thromboembolism or sudden death caused by aneurysm rupture, it is necessary to eliminate the aneurysm and that can be achieved safely by performing our recommended procedure. To establish safe and reliable bypasses, we use arterial grafts and create LAD and LCX bypasses using separate grafts. If the radial artery is not available, saphenous vein grafts (SVGs) can be used for the LCX region. LMT aneurysms near the bifurcation need to be fully excluded by the aggressive surgical strategy described in this study. Otherwise, there is still risk of enlargement and rupture of the residual aneurysm. In contrast, the use of an SVG for the entire LCX area carries a risk of global ischemia of this region related to vein graft disease in the long term and meticulous follow-up with antiplatelet therapy would be needed.

Although the option of percutaneous coronary intervention has become available in recent years, the use of PTFE-covered stents may be associated with the following complications: deployment of a covered stent in a CAA may result in occlusion of the branch arteries that originate within the aneurysm; incomplete coverage of the aneurysm may result in a persistent “leak” into the aneurysm sac; and PTFE-covered coronary stents pose a risk of thrombosis or in-stent restenosis. Stent length and aneurysm caliber (diameter > 10 mm) of PTFE-covered stents have also been reported as independent risk factors for future restenosis [16]

This study was limited by its retrospective nature and small patient cohort. Considering the rarity of CAAs, prospective studies to compare surgical exclusion and catheter-based interventions would not be feasible: however, an in-depth review of all cases and/or a meta-analysis would contribute to identifying the optimal treatment for this condition. A nationwide registry study would also be useful for improving the understanding of this pathology and exploring optimal treatments.

In conclusion, surgical exclusion of a CAA by full closure of the feeding proximal and the distal coronary arteries, supplemented with CABG using arterial conduits exclusively, is feasible and safe for patients with an idiopathic CAA in the proximal coronary arteries. Although further accumulation of such patients with long-term follow-up is needed, our data suggest that the aggressive surgical approach we described is a good option for treating this pathology.
